# Associations between tryptophan and iron metabolism observed in individuals with and without iron deficiency

**DOI:** 10.1038/s41598-019-51215-8

**Published:** 2019-10-10

**Authors:** Julian Wenninger, Andreas Meinitzer, Sandra Holasek, Wolfgang J. Schnedl, Sieglinde Zelzer, Harald Mangge, Markus Herrmann, Dietmar Enko

**Affiliations:** 1 Clinical Institute of Medical and Chemical Laboratory Diagnostics, Medical University of Graz, Auenbruggerplatz 15, 8036 Graz, Austria; 2 Department of Immunology and Pathophysiology, Medical University of Graz, Otto Loewi Research Center, Heinrichstraße 31a, 8010 Graz, Austria; 3 Practice for General Internal Medicine, Dr. Theodor-Körner-Straße 19b, 8600 Bruck/Mur, Austria; 4 Institute of Clinical Chemistry and Laboratory Medicine, General Hospital Hochsteiermark, Vordernbergerstraße 42, 8700 Leoben, Austria

**Keywords:** Diagnostic markers, Translational research

## Abstract

Current literature proposes associations between tryptophan metabolism and anaemia. However, study cohorts are rather small and final conclusions are still lacking. Here, we evaluated potential associations of tryptophan, kynurenine, and kynurenic acid with indicators of iron metabolism (i.e., mean corpuscular volume, mean corpuscular haemoglobin, ferritin, transferrin saturation, serum iron, transferrin, soluble transferrin receptor, reticulocyte haemoglobin) and haemoglobin in 430 individuals grouped by the presence or absence of iron deficiency or anaemia. Indicators of tryptophan metabolism were positively correlated with haemoglobin and markers of iron metabolism (p-values: <0.001–0.038; r-values: 0.100–0.305). The strongest correlation was observed between tryptophan and haemoglobin (p < 0.001, r = 0.305). The cubic regression model yielded the highest R-square values between haemoglobin and tryptophan markers. Overall, 115 patients with iron deficiency showed lower tryptophan and kynurenic acid concentrations compared to 315 individuals without iron deficiency. Six patients with anaemia of chronic disease were observed with the lowest serum tryptophan levels and the highest kynurenine/tryptophan ratio compared to 11 individuals with iron deficiency anaemia and 413 non-anaemic patients. This study showed little/moderate associations between haemoglobin, biomarkers of iron metabolism and tryptophan markers. Further studies are needed to get better insight in the causality of these findings.

## Introduction

In the last few years, the clinical interest in evaluating the tryptophan metabolism has increased. Tryptophan is an essential amino acid, which is utilized for protein biosynthesis and metabolized via the kynurenine pathway. It is also known as a precursor of the serotonin synthesis in the central nervous system. The enzyme indoleamine 2,3-dioxygenase, which is induced by proinflammatory cytokines (i.e., interferon-γ, tumor necrosis factor-α), converts tryptophan into kynurenine^1^. This degradation of tryptophan is expressed by the kynurenine/tryptophan ratio, which was first introduced by Fuchs *et al*.^2^ and which is able to assess the activity of the indoleamine 2,3-dioxygenase in the situation of an active immune systeme^3^.

Kynurenine is further metabolized into the neuroprotective kynuric acid and several neurotoxins^1^. The ratio between kynurenic acid and kynurenine enables the assessment of the neuroprotective index^3^. Since kynurenic acid hardly crosses the blood brain barrier, serum concentrations may not reflect the actual situation in the brain.

The kynurenine pathway as the primary route for tryptophan catabolism has received increasing attention as its dysregulation is considered to be associated with inflammation, tumor proliferation, neurodegenerative diseases and depression^4^. The tryptophan availability and metabolism have been reported to be associated with malabsorption conditions (i.e., fructose malabsorption) and mood disorders^5,6^. An increased indoleamine 2,3-dioxygenase activity with an enhanced degradation of tryptophan is also considered to be involved in the drop of blood levels of haemoglobin and the development of anaemia^7–10^.

Previous studies investigating possible associations between haemoglobin concentrations, anaemia and tryptophan metabolism were designed with rather small patient groups with anaemia of chronic disease (ACD)^7,11^ or performed as animal models with different degrees of renal insufficiency^8^. Furthermore, the question raises if the tryptophan metabolism has a potential link to iron deficiency or iron deficiency anaemia (IDA). This issue has been addressed by Weiss *et al*.^7^ but did not allow a convincing final conclusion at that time.

The heightened awareness of potential adverse effects (i.e. changes in cognitive development, immune function, energy metabolism or temperature regulation) of iron deficiency has renewed efforts to reduce the prevalence of this micronutrient deficiency^12^. Currently, there is no full international consensus on disease markers to be used for assessing the human iron status. Plasma ferritin levels (iron deficiency: <30 ng/mL) and transferrin saturation values (iron deficiency: <20%) remain the widespread biomarkers in defining iron deficiency in clinical practice^13,14^. Nevertheless, there is still a lack of study designs investigating the serum levels of tryptophan and tryptophan metabolites in large cohorts of patients with iron deficiency or anaemia.

The present study was conducted to investigate possible associations between the parameters of tryptophan and iron metabolism and haemoglobin levels in a large cohort of patients grouped by the presence or absence of iron deficiency or anaemia.

## Results

### Study population characteristics

The baseline characteristics of the study population are presented in Table 1. Of all 430 study participants, 290 (67.4%) were female, and 140 (32.6%) were male. The median age was 39 (range: 15–82) years.

**Table 1:** Baseline characteristics of the study population.

Study population (n = 430)	Min	Median	Max	Mean	SD*	Reference ranges
Age (y)	15	39	82	40.6	15.5	—
Height (cm)	139	169	198	170	9.4	—
Weight (kg)	20	70.5	149	72.9	17.1	—
Body mass index (kg/m^2^)	7.3	24.2	49.1	25.1	5.1	18.5–25.0
Haemoglobin (g/dL)	7.8	13.9	17.1	14	1.2	Males: 13.0–18.0 Females: 12.0–16.0
Mean corpuscular volume (fL)	66.9	86.2	98.1	86	4.3	80–99
Mean corpuscular haemoglobin (pg)	19.9	29.8	35.9	29.7	1.8	27–36
Ferritin (µg/L)	1	58	401	80.2	27–105	≥15
Transferrin saturation (%)	2	27	87	28.1	12.2	≥20
Iron (µg/dL)	6	101	264	103.5	40.2	65–175
Transferrin (g/L)	1.8	2.6	4.6	2.7	0.4	2–3,6
Soluble transferrin receptor (mg/L)	0.6	1.1	4.6	1.2	0.4	0.76–1.76
Reticulocyte haemoglobin (pg)	21.1	31.3	35.1	31.1	1.8	28–35
Tryptophan (µmol/L)	30.5	60.1	96.5	60.5	10.1	43–89
Kynurenine (µmol/L)	1.1	2.4	4.3	2.4	0.5	1.0–2.9
Kynurenic acid (nmol/L)	8.2	31.1	85.3	33.2	13.2	20–93
Kynurenine/tryptophan	0.02	0.04	0.08	0.05	0.01	—
Kynurenic acid/kynurenine	4	13.1	31.1	13.9	4.7	—
Creatinine (mg/dL)	0.3	0.7	1.2	0.7	0.1	Males: 0.7–1.3 Females: 0.55–1.02
eGFR (mL/min/1.73 m^2^)	60.6	105.4	160	105.5	17.8	>70
C-reactive protein (mg/L)	0	0	33	1	3.4	0–3

Kynurenine/tryptophan = tryptophan break-down index; kynurenic acid/kynurenine = neuroprotective ratio; eGFR, estimated glomerular filtration rate; min, minimum; max, maximum; SD, standard deviation. *In case of a skewed data distribution the 25^th^ and 75^th^ centiles are presented instead of SD.

Based on a ferritin <15 µg/L and/or a TSAT <20%, 115/430 (26.7%) individuals (97 females and 18 males) were found with iron deficiency. All in all, 17/430 (4%) patients (15 females and 2 males) had anaemia. Of these, 11 (64.7%) individuals were identified with IDA and 6 (35.3%) patients (5 females and 1 male) with ACD, respectively.

### Correlations between indicators of tryptophan and iron metabolism

The indicators of tryptophan metabolism correlated with iron metabolism. Serum tryptophan levels were positively correlated with haemoglobin (p < 0.001, r = 0.305) and ferritin (p = 0.038, r = 0.100). Kynurenine and kynurenic acid showed positive correlations with haemoglobin (p-values < 0.001, r = 0.176 and 0.296), soluble transferrin receptor (p = 0.007 and 0.038, r = 0.131 and 0.100), and ferritin (p-values < 0.001, r = 0.186 and 0.301), respectively.

### Assessment of the associations between soluble transferrin receptor and indicators of tryptophan metabolism

The univariate log-transformed linear regression model for the log-transformed soluble transferrin receptor is presented in Table 2. The tryptophan metabolism indicators kynurenine and kynurenine/tryptophan showed a statistically relevant influence on soluble transferrin receptor.

**Table 2:** Univariate log-transformed linear regression model for the log-transformed soluble transferrin receptor.

LOG Soluble transferrin receptor (mg/L)	ß-coefficient	p-value	95% CI	Percentage change
LOG Tryptophan (µmol/L)	0.007	0.882	−0.088–0.102	0.11%
LOG Kynurenine (µmol/L)	0.174	<0.001	0.081–0.268	2.02%
LOG Kynurenic acid (nmol/L)	0.068	0.157	−0.026–0.163	0.44%
LOG Kynurenine/tryptophan	0.153	0.001	0.059–0.247	1.61%
LOG Kynurenic acid/kynurenine	−0.034	0.482	−0.129–0.061	−0.25%

The column “percentage change’’ represents the increase/decrease of soluble transferrin receptor under the assumption of a 10% increase of the independent variables. CI, confidence interval.

### Assessment of the associations between ferritin and indicators of tryptophan metabolism

The univariate log-transformed linear regression model for log-transformed ferritin estimating crude effects (model 1) and adjusted for the C-reactive protein (model 2) is demonstrated in Table 3. The tryptophan metabolism indicators kynurenic acid and kynurenic acid/kynurenine showed a statistically relevant influence on ferritin. Adjusted for the C-reactive protein, the association between ferritin and the tryptophan markers stayed similar.

**Table 3:** Univariate log-transformed linear regression models for log-transformed ferritin estimating crude effects (model 1) and adjusted for inflammation (C-reactive protein) (model 2).

LOG Ferritin (µg/L)	ß-coefficient	p-value	95% CI	Percentage change
**Model 1: crude**				
LOG Tryptophan (µmol/L)	0.105	0.029	0.011–0.200	6.45%
LOG Kynurenine (µmol/L)	0.144	0.003	0.050–0.238	6.66%
LOG Kynurenic acid (nmol/L)	0.272	<0.001	0.180–0.363	7.05%
LOG Kynurenine/tryptophan	0.059	0.221	−0.036–0.154	2.43%
LOG Kynurenic acid/kynurenine	0.221	<0.001	0.128–0.313	6.60%
**Model 2: adjusted for C-reactive protein**				
LOG Tryptophan (µmol/L)	0.110	0.026	0.013–0.207	6.76%
LOG Kynurenine (µmol/L)	0.147	0.003	0.052–0.242	6.77%
LOG Kynurenic acid (nmol/L)	0.274	<0.001	0.183–0.366	7.12%
LOG Kynurenine/tryptophan	0.063	0.210	−0.035–0.161	2.58%
LOG Kynurenic acid/kynurenine	0.230	<0.001	0.136–0.325	6.91%

The column “percentage change“ represents the increase of ferritin under the assumption of a 10% increase of the independent variables. CI, confidence interval.

### Tryptophan metabolism measurements in individuals with iron deficiency

**Figure 1:**
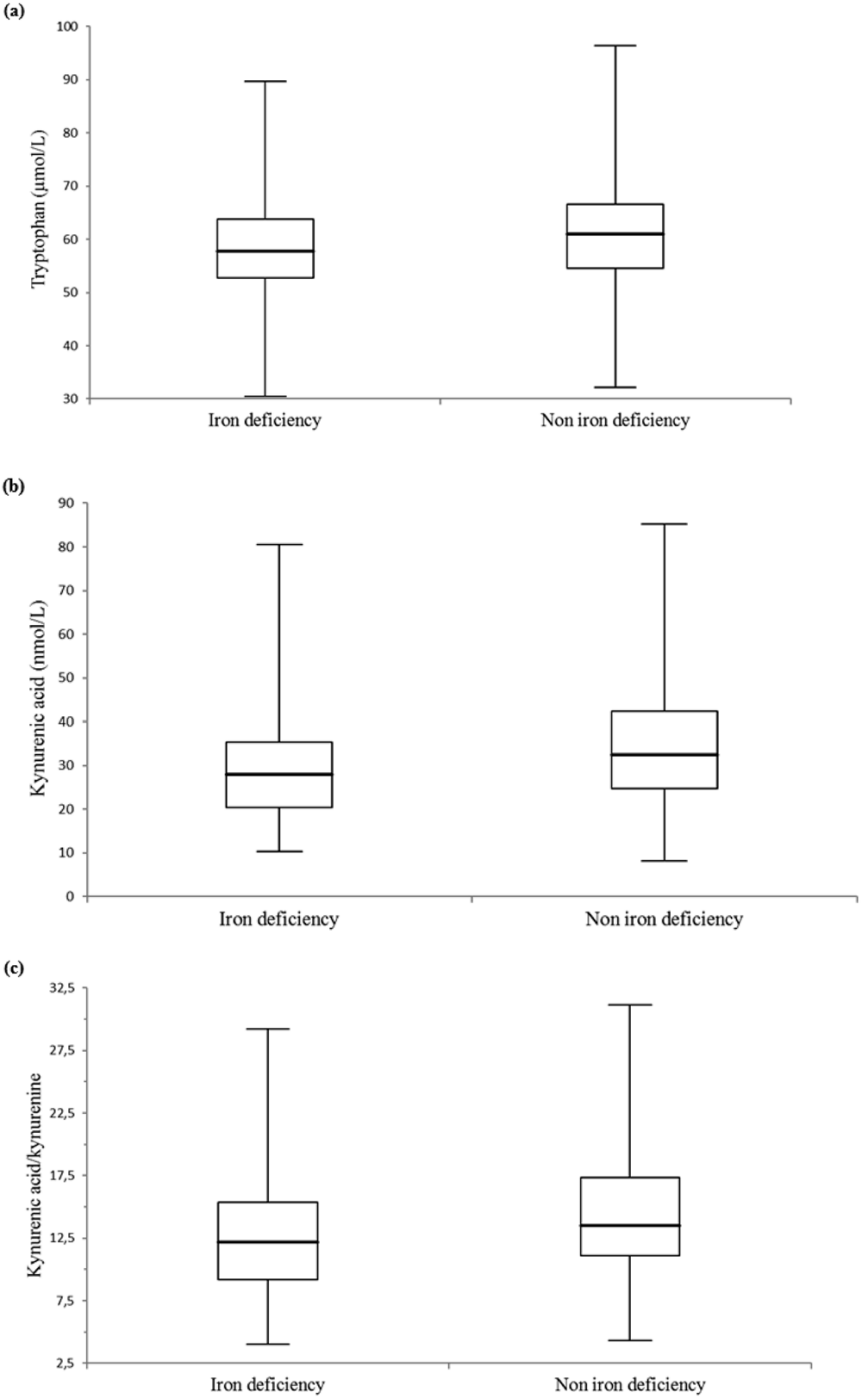
Tryptophan metabolism and iron deficiency. (**a**) Tryptophan, (**b**) kynuric acid, and (**c**) neuroprotective ratio (kynurenic acid/kynurenine) comparisons between 115 and 315 individuals with and without iron deficiency (p-values were 0.005 for tryptophan and <0.001 for kynurenic acid and kynurenic acid/kynurenine). The central boxes represent the 25^th^ to 75^th^ percentile range. The lines inside the boxes show the median value for each group. Minimum and maximum are indicated as whiskers with end caps.

Overall, 115 patients with iron deficiency had different tryptophan levels (57.8 [52.7–63.8] vs. 61.0 [54.6–66.5] μmol/L, p = 0.005) and kynurenic acid serum levels (27.9 [20.4–35.3] vs. 32.5 [24.7–42.4] μmol/L, p < 0.001), and also different kynurenic acid/kynurenine index levels (12.2 [9.2–15.4] vs. 13.5 [11.1–17.4], p = <0.001) compared to 315 individuals without iron deficiency, respectively. The box-and-whisker plots of tryptophan, kynurenic acid and kynurenic acid/kynurenine for patients with and without iron deficiency are illustrated in Fig. 1(a–c).

### Tryptophan metabolism in anaemic patients

**Figure 2:**
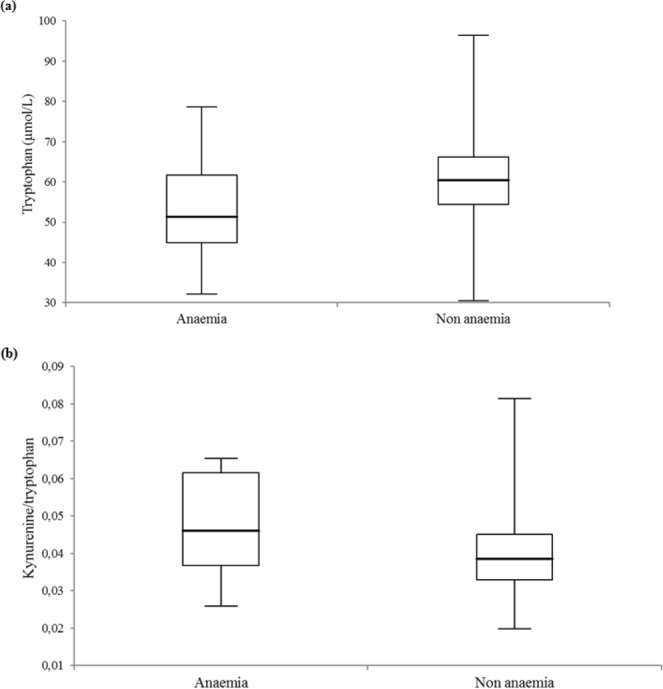
Tryptophan metabolism and anaemia. Box-and-whisker plots of (**a**) tryptophan serum level and (**b**) tryptophan break-down index (kynurenine/tryptophan) comparisons between 17 anaemic and 413 non-anaemic individuals (p-values were 0.003 and 0.012). The central boxes represent the 25^th^ to 75^th^ percentile range. The lines inside the boxes show the median value for each group. Minimum and maximum are indicated as whiskers with end caps.

As shown in Fig. 2a,b, patients with anaemia had lower median tryptophan serum levels (51.3 [44.9–61.7] vs. 60.5 [54.5–66.2] μmol/L, p = 0.003) and a higher kynurenine/tryptophan ratio (0.05 [0.04–0.06] vs. 0.04 [0.03–0.005], p = 0.012).

The univariate log-transformed linear regression model for the assessment of associations between haemoglobin and indicators of tryptophan metabolism is demonstrated in Table 4. Additionally, we fitted quadratic, cubic, exponential, fractional polynomial, and cubic spline regression models for possible non-linear relationships. The cubic regression model yielded the highest, yet relatively small, R-square values (R^2^ = 0.101 for tryptophan, R^2^ = 0.028 for kynurenine, R^2^ = 0.080 for kynurenic acid, R^2^ = 0.027 for kynurenine/tryptophan, and R^2^ = 0.064 for kynurenic acid/kynurenine), indicating little to moderate associations between haemoglobin and tryptophan indicators despite the small p-values (p < 0.001 for tryptophan, kynurenic acid and kynurenic acid/kynurenine, p = 0.007 for kynurenine, and p = 0.008 for kynurenine/tryptophan).

**Table 4:** Univariate log-transformed linear regression model for log-transformed haemoglobin.

LOG Haemoglobin (g/dL)	ß-coefficient	p-value	95% CI	Percentage change
LOG Tryptophan (µmol/L)	0.305	<0.001	0.214–0.395	1.59%
LOG Kynurenine (µmol/L)	0.107	0.027	0.012–0.201	0.42%
LOG Kynurenic acid (nmol/L)	0.261	<0.001	0.170–0.353	0.57%
LOG Kynurenine/tryptophan	−0.111	0.021	−0.206 – −0.017	−0.39%
LOG Kynurenic acid/kynurenine	0.233	<0.001	0.141–0.325	0.59%

The column “percentage change“ represents the increase/decrease of haemoglobin under the assumption of a 10% increase of the independent variables. CI, confidence interval.

**Figure 3:**
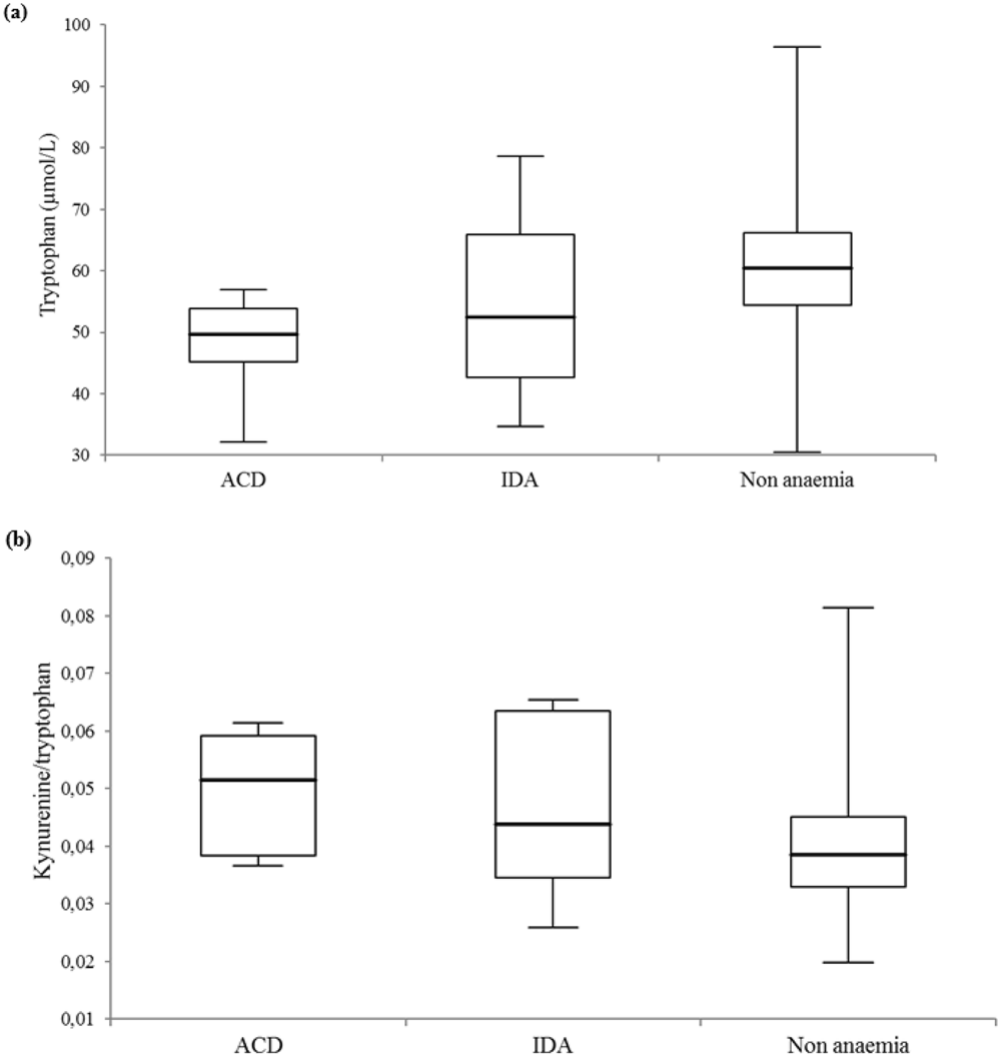
Tryptophan metabolism and categories of anaemia. Box-and-whisker plots of (**a**) tryptophan serum level and (**b**) tryptophan break-down index (kynurenine/tryptophan) comparisons between 6 and 11 individuals with anaemia of chronic disease (ACD) and iron deficiency anaemia (IDA) and 413 non-anaemic subjects (p-values were 0.004 and 0.031). The central boxes represent the 25^th^ to 75^th^ percentile range. The lines inside the boxes show the median value for each group. Minimum and maximum are indicated as whiskers with end caps.

Markers of tryptophan metabolism categorized by types of anaemias (ACD and IDA) are illustrated in Fig. 3(a,b). Six patients with ACD (n = 6) showed the lowest median tryptophan serum levels (49.6 [45.2–53.9] μmol/L) and the highest kynurenine/tryptophan ratio (0.05 [0.04–0.06]) compared to 11 individuals with IDA (tryptophan: 52.4 [42.7–65.9] μmol/L; kynurenine/tryptophan: 0.04 [0.03–0.06]) and 413 non-anaemic patients (tryptophan: 60.5 [54.5–66.2] μmol/L; kynurenine/tryptophan: 0.04 [0.03–0.05]) (p- values were 0.004 and 0.031), respectively.

## Discussion

We found positive correlations between the serum tryptophan, kynurenine, kynurenic acid concentrations and haemoglobin and ferritin levels. The strongest association was observed between the tryptophan and haemoglobin concentrations (p < 0.001, r = 0.305). The cubic regression model showed the highest, yet relatively small, R^2^-values, indicating little/moderate associations between haemoglobin and indicators of tryptophan metabolism.

Lowered serum tryptophan levels were found with lowered haemoglobin values. A 10% increase of tryptophan was shown to be associated with a 1.59% increase of haemoglobin. The tryptophan availability is a limiting factor for protein biosynthesis and cell growth and is also of importance for erythropoiesis^7^. Tryptophan is a nutritional pyrrole source, which is essential for the haemoglobin synthesis. Human haemoglobin contains 6 tryptophan residues, which are located in the α- and β-subunits of the haemoprotein^15–17^. Recently, a cross-sectional study, which included 105 South African HIV-infected patients and 60 HIV-negative controls, reported a positive correlation (p < 0.001, r = 0.378) between the nutritional indicators haemoglobin and tryptophan^18^. Malnutrition may be one possible reason for tryptophan depletion. The required minimum daily intake of tryptophan is estimated with 175 mg for adult women and 250 mg for adult men^18–20^.

Present data show that tryptophan serum levels correlated with the iron storage protein ferritin (p = 0.038, r = 0.100). The correlation coefficient was relatively small indicating only a weak correlation. A 10% increase of tryptophan was shown to be associated with a 6.45% increase of ferritin. Individuals with iron deficiency (ferritin <15 µg/L and/or a TSAT <20%) were found with lowered tryptophan concentrations. In a previous study, apoferritin, the hollow shell protein component of ferritin, was shown to contain tryptophan residues^21^. This could be one possible reason for the positive correlation between tryptophan and ferritin found here.

Another possible explanation for the observed relationship between tryptophan and iron metabolism could be that both metabolic pathways may be influenced by acute or chronic inflammatory conditions. Ferritin is known as an acute phase reactant, which can result in hypoferremia due to iron sequestration in the reticuloendothelial macrophages with subsequent iron-restricted erythropoiesis^22^. In a previous published study, lung cancer patients with inflammation and immune activation were shown to have lower tryptophan serum levels and an increased kynurenine/tryptophan ratio, indicating that the kynurenine pathway activation is accelerated under inflammatory conditions^11^.

Here, patients with anaemia were found with lower serum tryptophan levels and a distinct increased tryptophan breakdown index compared to non-anaemic individuals. This finding is in agreement with an earlier study by Weiss *et al*., which reported lower tryptophan concentrations in patients with ACD compared to healthy controls^7^. Differences between the study designs must be mentioned. Weiss *et al*. studied 22 hospitalized patients with a variety of chronic diseases and ACD (median age 78 years) and 22 non-anaemic controls (median age 75 years)^7^. In comparison, we recruited 430 ambulatory individuals (median age 39 years) without chronic diseases in their case histories for haemoglobin and iron status assessment. In our cohort, we differentiated between ACD and IDA and observed the lowest tryptophan concentrations and the highest kynurenine/tryptophan ratio in patients with ACD compared to individuals with IDA and non-anaemic subjects.

We used Thomas-Plot analyses for the differential diagnosis of IDA and ACD. Previous studies demonstrated a good analytical performance of this diagnostics approach^23,24^. Present data showed relatively few ACD cases in this study population. One possible explanation of this phenomenon could be the fact that the cohort consisted mostly of young adults without relevant comorbidities.

An accelerated tryptophan catabolism appears to be involved in the pathogenesis of ACD. Cytokine-induced (i.e., interferon-γ, tumor necrosis factor-α) tryptophan degradation via the enzyme indoleamine 2,3-dioxygenase suppresses the erythropoiesis^25^. As a consequence, the enhanced tryptophan breakdown to kynurenine results in a deficiency of this essential amino acid^7^. Given that tryptophan is known to bind non-covalently to the negative acute-phase protein albumin^26^, an inflammation-induced reduction in albumin concentrations could also have a potential influence on the serum tryptophan concentrations.

The current problem is, that all these observations were made in cross-sectional studies, which cannot serve as a basis for a cause-effect conclusion^27^. Therefore, all these considerations should be extended in further longitudinal prospective studies. In addition, the potential link between tryptophan, haemoglobin and iron parameters found in the present work could be subject of a basic research study, which covers the tryptophan metabolism of erythrocytes. This approach might have potential to gain better insight into the interaction of the metabolic tryptophan pathway and the erythropoiesis.

The major limitation of this cross-sectional study is that pro-inflammatory cytokines (i.e., interferon-γ, tumor necrosis factor-α) and the acute-phase reactant α1-acid-glycoprotein were not measured.

In conclusion, little/moderate associations between haemoglobin, biomarkers of iron metabolism and tryptophan indicators were observed. Patients with iron deficiency or anaemia showed lower serum tryptophan levels compared to individuals without iron deficiency or anaemia.

## Materials and Methods

### Study design and patients

A total of 430 consecutive patients, who were admitted by general practitioners and specialists to the outpatient clinic for a medical check-up of their actual iron status, were included in this cross-sectional study. All participants provided their written informed consent. They underwent venous blood sampling after an overnight fasting state in the morning (between 8.00 and 10.00 a.m.). The samples were used to investigate the iron metabolism (i.e., haemoglobin, mean corpuscular volume, mean corpuscular haemoglobin, ferritin, transferrin saturation, serum iron, transferrin, soluble transferrin receptor, reticulocyte haemoglobin), the tryptophan metabolism (tryptophan, kynurenine, kynurenic acid, kynurenine/tryptophan ratio, kynurenic acid/kynurenine index), the renal function (creatinine, estimated glomerular filtration rate [eGFR]), and the C-reactive protein.

The inclusion criteria for this study were referral to our outpatient clinic for actual iron status assessment, minimum age of 15 years, and performance of laboratory analyses within 4 hours after blood sampling. Patients with oral or intravenous iron supplementation within the last year before the study and/or with unavailability of all study parameters were excluded from the study. Approval was obtained from the Ethical Committee of the Johannes Kepler University Linz (Linz, Austria). The study was carried out according to the latest version of the Declaration of Helsinki.

### Blood collection and laboratory procedures

From all 430 study participants, one 2 mL VACUETTE^®^ EDTA tube was collected for haemoglobin, mean corpuscular volume, mean corpuscular haemoglobin, and reticulocyte haemoglobin measurements, one 4 mL VACUETTE^®^ LH lithium tube for ferritin, transferrin, iron, soluble transferrin receptor, and C-reactive protein analyses, and one 4 mL VACUETTE^®^ Z Serum Sep Clot Activator tube (all tubes were from Greiner Bio-one International GmbH) for tryptophan, kynurenine, and kynurenic acid measurements. Plasma and serum samples were centrifuged at 2000 × g for 10 minutes at room temperature.

The determination of haemoglobin (anaemia: men: <13 g/dL; women: <12 g/dL), mean corpuscular volume (reference range: 80–99 fL), mean corpuscular haemoglobin (reference range: 27–36 pg) and reticulocyte haemoglobin (reference range: 28–35 pg) was performed on an ADVIA^®^ 2120i Hematology Analyzer (Siemens, Vienna, Austria). Ferritin (iron deficiency: <15 µg/L) was measured by chemiluminescent technology, serum iron (reference range: 65–175 µg/dL) by a bichromatic photometry method, and transferrin (reference range: 2–3.6 g/L), soluble transferrin receptor (reference range: 0.76–1.76 mg/L) and C-reactive protein (0–3 mg/L) by nephelometry on a Dimension Vista^®^ 1500 System (Siemens, Vienna, Austria). Iron deficiency was defined as a plasma ferritin level <15 µg/L and/or a transferrin saturation <20%^14,28,29^.

The biomarkers of tryptophan metabolism (i.e., tryptophan, kynurenine and kynurenic acid) were determined by high-performance liquid chromatography with a simultaneous ultraviolet and fluorimetric detection system^30^. In brief, 100 µL plasma sample was deproteinized by adding 100 µL of 5% (v/v) perchloric acid. After vortexing and 5 min centrifugation at 11,000 × g, 20 μL of the clear supernatant was injected into the chromatographic system. Separations were achieved on a Chromolith RP18e column (100 × 4.6 mm, 5 µm, Merck Darmstadt, Germany) at 30 °C by isocratic elution with a mobile phase (pH 4.9) consisted of 50 mmol/L ammonium acetate, 250 mol/L zinc acetate and 3% (v/v) acetonitrile, at a flow-rate of 0.8 mL/min. Kynurenine and tryptophan were detected on an Agilent 1200 VWD detector (Agilent, Palo Alto, CA, U.S.A.) at 235 nm, kynurenic acid was detected fluorometrically on an Agilent 1260 FLD detector. The acquisition and processing of the chromatograms were performed using an Agilent 1200 system equipped with a Chemstation software (Agilent, Palo Alto, CA, U.S.A.). All reagents were p.A. grade from Merck (Darmstadt, Germany). The intra-assay coefficients of variations (CVs) for different concentrations varied between 0.7–2.9% for tryptophan, 1.7–4.3% for kynurenine, and 2.6–4.5% for kynurenic acid. The inter-assay CVs ranged between 6.3–9%, 2.0–5.4%, and 8.4–11.6% for tryptophan, kynurenine, and kynurenic acid, respectively. The reference ranges of the tryptophan metabolites and the kynurenine/tryptophan ratio and neuroprotective kynurenic acid/kynurenine index were calculated according to the literature^3,31,32^.

Creatinine (males: 0.7–1.3 mg/dL; females: 0.55–1.02 mg/dL) was measured using an enzymatic method applied on a Roche Cobas Mira (Roche Diagnostics, Vienna, Austria). The eGFR (normal: >70 mL/min/1.73 m^2^) was calculated applying the Chronic Kidney Disease Epidemiology Collaboration (CKD-EPI) equation^33^.

### Statistics

All parameters were recorded in a descriptive statistical manner, tabulated and evaluated. Not normally distributed continous variables were presented as medians with interquartile ranges (Q1–Q3). Categorical variables were expressed as percentages.

For the comparison between two groups for not normally distributed continuous variables (verification with the Kolmogorov-Smirnov test with Lilliefors correction at a type-I error-rate of 10%) the Mann-Whitney U test was used. For the comparison between three or more groups for not normally distributed continuous variables (verification with the Kolmogorov-Smirnov test with Lilliefors correction at a type-I error-rate of 10%) the Kruskal-Wallis-test was performed.

To assess potential correlation between two continuous variables the Spearman’s rank correlation coefficient (not normally distributed data) was used. Furthermore, regression models (linear, log-linear, quadratic, cubic, exponential, fractional polynomial, cubic spline) were performed to assess the association between variables. The type I error was set to 5% (two-sided) without adjustment for multiple testing, so all p-values are only descriptive.

The percentage of transferrin saturation was calculated based on the formula: *transferrin saturation* (%) = *serum iron* (*µg/dL*) × *70*.*9/transferrin* (*mg/dL*). The differential diagnosis of IDA and ACD was based on Thomas-Plot analyses as described previously^23,24,29^. In brief: for the discrimination of the IDA and ACD the cut-off for the soluble transferrin receptor/log ferritin ratio depended on the acute-phase reaction and was 1.5 for a C-reactive protein ≤5 mg/L and 0.8 for a C-reactive protein >5 mg/L, respectively.

For statistical analysis, the statistical computing software R Version 3.5.2 (R Foundation for Statistical Computing, Vienna, Austria. http://www.R-project.org) including the standard-package splines respectively the optional package mfp (version 1.5.2) and the Analyse-it® software version 4.92 (Analyse-it Software, Ltd., Leeds, United Kingdom) were used.

## Data Availability

The datasets generated and analysed during the current study are available from the corresponding author on reasonable request.
